# A Rare Complication of Bladder Injury Following Laparoscopic Appendectomy: A Case Report and Literature Review

**DOI:** 10.7759/cureus.102852

**Published:** 2026-02-02

**Authors:** Amaar Aamery, Salim AlMaashani, Salim Tabook, Suma Jacob

**Affiliations:** 1 Department of General Surgery, Sultan Qaboos Hospital, Salalah, OMN; 2 Department of Radiology, Sultan Qaboos Hospital, Salalah, OMN

**Keywords:** appendicitis, bladder catheterization, bladder injury, ct cystogram, laparoscopic appendectomy, surgical complication, trocar injury, urinary bladder injury

## Abstract

Acute appendicitis is widely recognized as the leading cause of acute abdominal pain in patients seeking hospital treatment. The standard of care and golden approach has traditionally involved performing an appendicectomy, which nowadays is more often performed using laparoscopic techniques. As with any laparoscopic procedure, there is a risk of iatrogenic organ injury, particularly during trocar insertion; one such organ at risk is the urinary bladder (0.17-0.73%). Most reported cases of urinary bladder injuries occur due to trocar insertion in the suprapubic area. In this report, we present a case of a patient with an occult bladder injury diagnosed postoperatively on day 3 via creatinine level in drain fluid after undergoing a laparoscopic appendicectomy. We also include a review of the literature regarding the presentation, diagnosis, and management of this complication.

## Introduction

Acute appendicitis is recognised globally as the leading cause of acute abdomen, with an incidence of approximately 90-100 new cases per 100,000 individuals annually [1]. The standard of care and golden approach has been and still is to perform an appendicectomy. Traditionally performed via the muscle-splitting incision described by (and named after) McBurney, appendicectomy is performed more often laparoscopically today. Several trials and meta-analyses have shown decreased pain, lower morbidity, shorter hospital stay, quicker return to normal activities, and better cosmesis via this approach [2]. The complication rate for laparoscopic appendicectomy historically has ranged from 6.71% to 12.7%, with the most common complications being surgical site/deep wound infections, ileus, and intra-abdominal bleeding. As with any laparoscopic procedure, there is a rare risk of iatrogenic organ injury, particularly during trocar insertion [3]. As described, iatrogenic injuries during laparoscopic appendicectomy are rare. There is minimal data on iatrogenic urinary bladder injuries during laparoscopic appendicectomy, as these types of injuries are more common with laparoscopic hernia and gynaecological surgeries. Most reported injuries to the urinary bladder are related to trocar insertion at the suprapubic region, as the filled adult urinary bladder can even reach the umbilicus. The incidence of such injuries has been estimated to be at 0.36%. Urinary bladder injuries can be diagnosed immediately at the time of surgery with observation of urinary leak (especially in a non-catheterised distended urinary bladder) or can be an occult injury that is diagnosed only postoperatively. Diagnosis of such injuries postoperatively can be challenging and includes fluid assessment (if any drain is left) for creatinine levels and CT cystography to assess the severity of the injury and to inform the choice of management approach. Some literature recommends urethral catheterisation to prevent such injuries. However, others have reported urinary bladder injury despite ‘in-and-out’ catheterisation to empty the urinary bladder preoperatively [4]. We describe a patient who underwent a laparoscopic appendicectomy for acute appendicitis, which was complicated by an occult urinary bladder injury, and the method of diagnosing such injuries and highlighting the success of the conservative approach for management of urinary bladder injury.

## Case presentation

Our patient is a 20-year-old male who presented with a one-day history of abdominal pain that started at the right iliac fossa, which worsened, and he vomited once. He sought advice at our emergency department. He denied any previous similar history, and his pain did not shift or radiate. He gave a history of nausea and loss of appetite. He denied any urinary symptoms and no chronic medical illness, nor has he had any surgical history. On examination, his vitals were normal, and he was afebrile. He was found to have pain in the lower abdomen, mainly on the right side, with guarding, and his Rovsing sign was positive.

His blood investigations revealed a white cell count (WCC) of 15 x 103/uL (2.2-10) and C-reactive protein (CRP) of 111 mg/L (0-5), while the rest were all within normal limits.

Based on the clinical presentation, examination findings, and the blood results, a diagnosis of acute appendicitis was made, and the patient was taken for diagnostic laparoscopy and appendicectomy. Surgery was done laparoscopically in standard technique with three trocars (one 11 mm at the infraumbilical location and two 5 mm) (Applied Medical, California, USA). Open access was achieved using the Hasson technique, and pneumoperitoneum was established at a pressure of 12 mmHg. Two additional trocars were introduced under direct vision, in the suprapubic midline position and the left iliac fossa in the midclavicular position, for triangulation. Findings were of a gangrenous, friable, thick appendix, pre-ileal in position, forming an early mass wrapped up in omentum, with free pus in the pelvis. The mass was bluntly dissected, and the meso-appendix was diathermized and the base controlled with an ENDOLOOP^TM^ (Ethicon, Ohio, USA). Drain F14 (Romsons, Brussels, Belgium) was left in the pelvis and exteriorised through the left iliac fossa trocar site.

**Figure 1:**
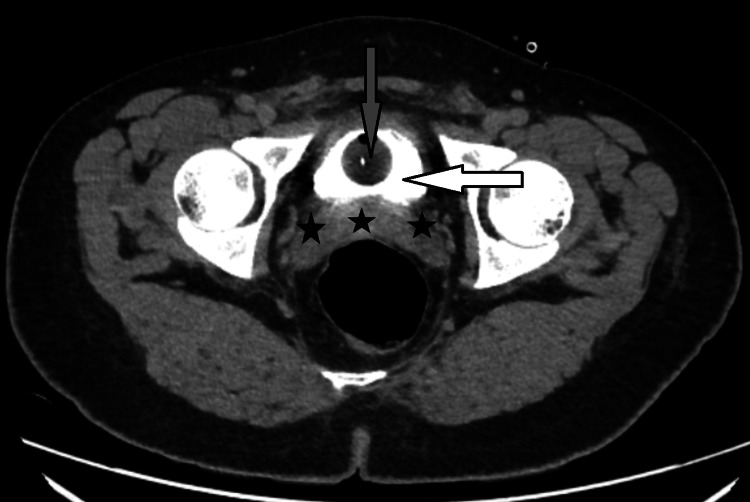
CT abdomen axial view (10-minute delay sequence) day 3 after surgery showing contrast in the bladder (white arrow) with no contrast leak in the peri-vesical area (black stars) with a Foley catheter (gray arrow) in place. White arrow: urinary bladder with contrast, gray arrow: Foley catheter, black stars: peri-vesical area with no contrast leak CT: computerized tomography

**Figure 2:**
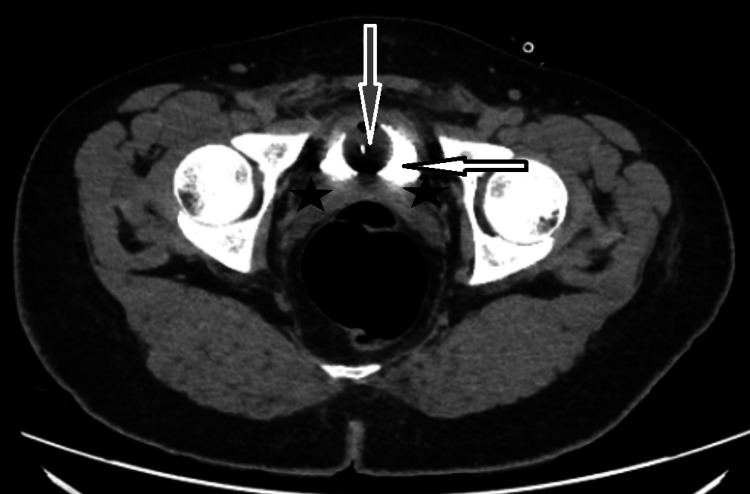
CT abdomen axial view (20-minute delay sequence) day 3 after surgery showing the bladder with contrast (white arrow) and a Foley catheter (grey arrow) in place with no contrast leak in the peri-vesical area (black stars). White arrow: urinary bladder with contrast, gray arrow: Foley catheter, black stars: peri-vesical area with no contrast leak CT: computerized tomography

Postoperatively, the patient was doing well, except for lower abdominal discomfort. On the postoperative day 1 (POD 1), his blood results showed improved WCC at 13.11 x 103/uL (2.2-10) and CRP at 69.23 mg/L (0-5). His drain was only 15 ml of serosanguinous fluid. However, on POD 2, the amount of serous fluid drained increased to 55 ml, and on POD 3, it increased to 220 ml. At that point, the diagnosis of a missed bladder injury was suspected and confirmed by the drain fluid analysis showing a creatinine level of 11354 umol/L (3,450-22,900) compared to the patient's serum creatinine level of 69 umol/L (45-100). A Foley^TM^ catheter (Microtech, Mönchengladbach, Germany) was inserted, and a CT abdomen-pelvis was arranged. CT showed no apparent contrast leak from the ureters or bladder on the delayed phase, with minimal free fluid in the pelvis, as shown in Figures 1-2. The CT findings support the diagnosis of a minor small bladder injury that sealed, as it did not show any leak after FOLEY catheter insertion, supporting the conservative management approach in our patient.

**Figure 3:**
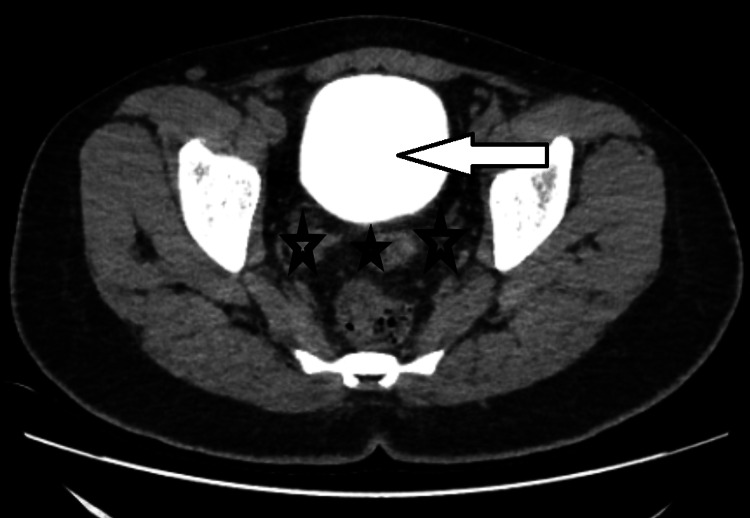
CT cystogram axial view on day 10 after surgery (supine position) showing contrast in the bladder (white arrow) with no contrast leak in the peri-vesical area (black stars). White arrow: urinary bladder with contrast, black stars: peri-vesical area with no contrast leak CT: computerized tomography

**Figure 4:**
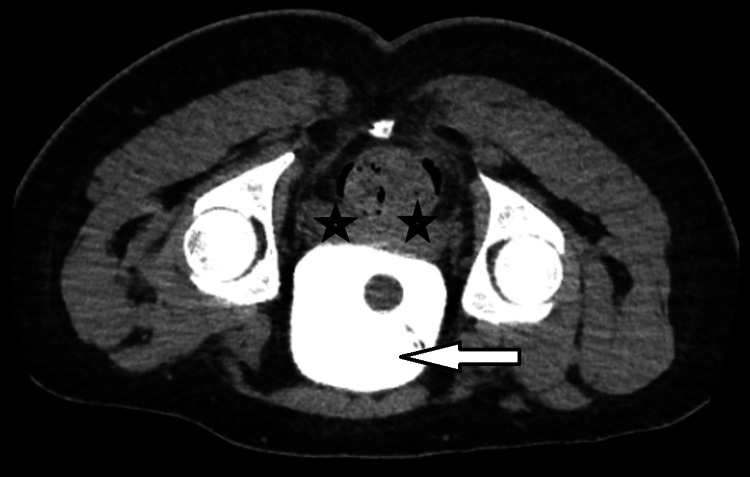
CT cystogram axial view 10 days after surgery (prone position) showing the bladder full of contrast (white arrow) with no contrast leak in the peri-vesical area (black stars). White arrow: urinary bladder with contrast, black stars: peri-vesical area with no contrast leak CT: computerized tomography

The urology team was involved and agreed to a conservative management approach with a Foley catheter for 10 days. We explained the unfortunate complication to the patient and, based on the CT findings, reassured him that conservative management should suffice. The drain was removed by the surgical team on POD 5 after it stopped draining, and the patient was discharged home for outpatient follow-up, with a plan for repeat CT cystogram before Foley catheter removal on POD 10. The patient was reviewed in the clinic on POD 10 after surgery, and a CT cystogram was performed, which showed the urinary bladder well distended with the Foley catheter bulb in situ and no contrast leak in supine/prone positions, as shown in Figures 3-4. Based on that, the Foley catheter was removed, and the patient was reviewed a week later (POD 17). The patient was doing well, with healed wounds and no urinary problems; therefore, they were discharged from the clinic.

## Discussion

​​​​​​As discussed, urinary bladder injuries in laparoscopic appendicectomies are rare complications. Levy et al. reported six iatrogenic urinary bladder injuries in a series of 1,671 laparoscopic appendicectomies [5]. In another case review, Joseph et al. reported a slightly lower rate of 0.17% in a series of 1147 laparoscopic appendicectomies [3]. The risk of urinary bladder injury in paediatric surgery is higher than in adults due to the comparatively smaller operative space, and the tendency of the full bladder to become an abdominal organ in the infant patient [6].

Iatrogenic bladder injury during laparoscopic appendicectomy happens mainly due to the suprapubic trocar insertion. Although not reported in the literature, it can theoretically occur due to intraoperative manipulation of laparoscopic instruments (diathermy and sharp instruments) during introduction through left iliac fossa ports, not under direct vision. In our case, the most probable cause would be a partial tangential urinary bladder injury at the time of the suprapubic port insertion, which didn’t show a urine leak at the time of port removal under vision.

Joseph et al. have highlighted that factors that could increase the risk of urinary bladder injury include anatomical variations, congenital anomalies such as Uracus, and the surgeon's training level. Other suggested factors can include improper supra-pubic trocar insertion, inadequate bladder emptying preoperatively (bladder functional disease or outlet obstruction), previous abdominal surgeries with intraoperative adhesions, and complicated pelvic appendicitis with mass formation [3].

**Figure 5:**
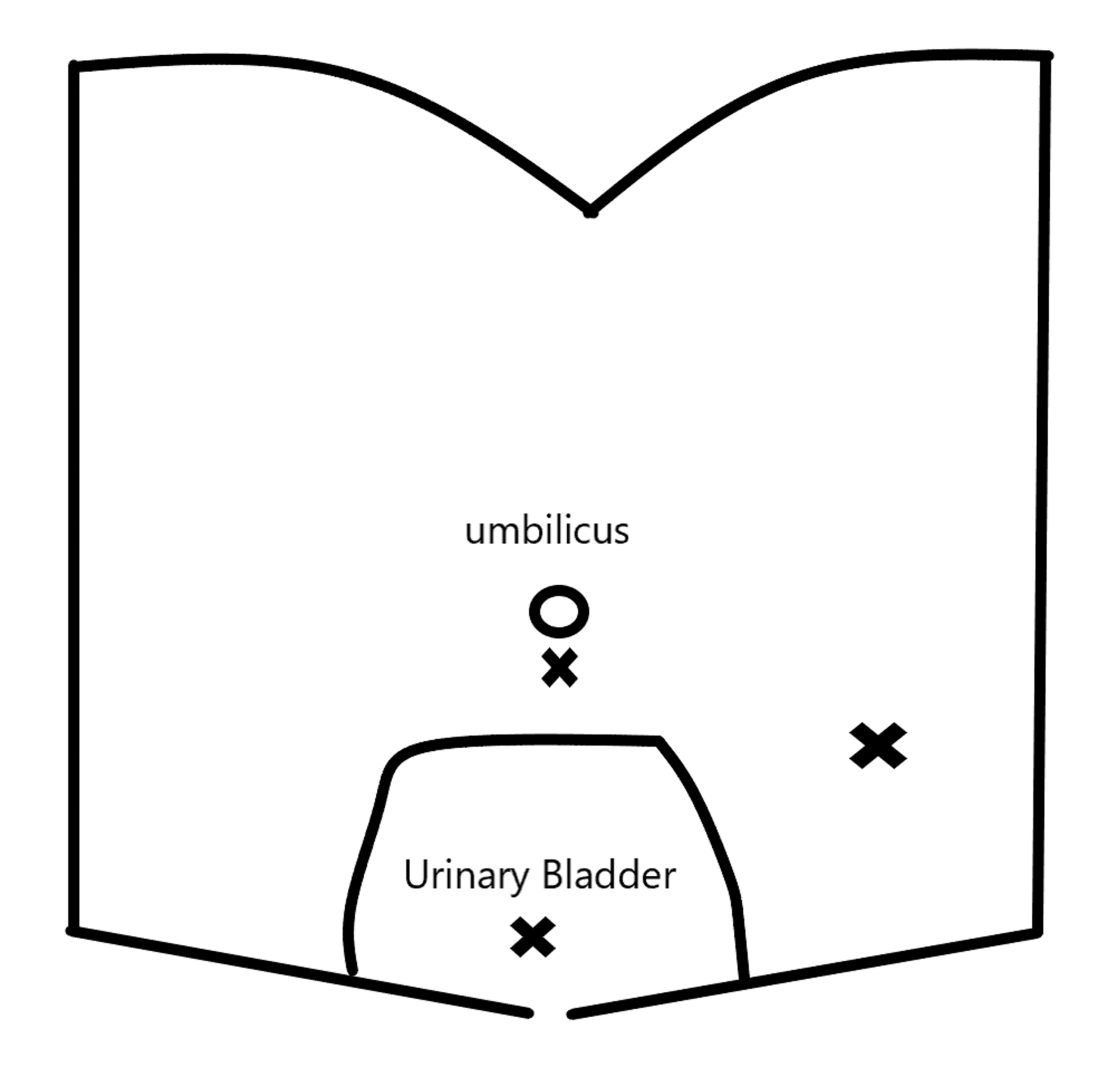
Port sites (X shapes) triangulation in laparoscopic appendicectomy showing the supra-pubic port location in relation to filled adult urinary bladder. X: three port sites: upper middle: infraumbilcal port, lower middle: suprapubic port, left side: left iliac fossa port

Careful consideration and laparoscopic identification of the urinary bladder before suprapubic trocar insertion isimportant for reducing the risk of these injuries. Selecting the anatomical site for the suprapubic catheter is also important. Despite the appeal of hiding a 5 mm port site scar in the pubic hairline, careful attention must be paid to the point of insertion [6]. While it is important to achieve optimal triangulation for safe surgery, placing the suprapubic port too close to the symphysis pubis increases the risk of bladder injury, which can reach nearly the level of the umbilicus when full in an adult patient, as shown in Figure 5.

There has been debate about the best approach to ensure bladder emptying preoperatively. This can be achieved by asking the patient to void on call to theatre without catheterisation, thereby reducing the risk of urinary tract injury since no instrumentation is performed, but increasing the risk of incomplete bladder emptying. Incomplete voiding may occur due to pain or bladder irritation from appendicitis, pre-existing bladder outlet obstruction, or detrusor failure [3]

Another approach to empty the urinary bladder is catheterisation, which can be either an in-and-out procedure with indwelling catheter insertion or leaving the FOLEY catheter in place throughout the surgery. The use of an indwelling versus a Foley catheter will increase the chances of full bladder emptying, but it comes with a higher risk of urinary tract infections and urethral injuries (more with foley catheter due to the inflatable balloon). Levy et al have reported only 16.67% risk reduction of bladder injuries after catheter insertion, indicating that urinary bladder catheterisation may not be enough to prevent bladder injuries completely [5].

Diagnosis of the urinary bladder injury can be either Immediately on table intraoperatively with noticed urine leak on removal of the suprapubic trocar under vision, or an Occult injury which can present early in the first day after surgery (with larger injuries), or delayed within three to four days post-surgery with patients coming back from home with abdominal pain and oligo or anuria [5]. Diagnosis of occult injuries in early or delayed presentations can be based on clinical symptoms and signs, blood investigation results, and drain fluid analysis and confirmed with radiological imaging. Symptoms such as unexpected worsening of abdominal pain after surgery, a large volume draining from the abdominal drain (which can be tested for urea/creatinine levels), and oliguria or anuria will be the first clues to suspect a urinary bladder injury. Blood investigations showing worsening WCC, CRP, or kidney function test (KFT) can also be associated, but are nonspecific. More importantly, biochemical analysis of the drain fluid, specifically the creatinine level, and comparison with the patient’s serum creatinine level can further support the diagnosis. The gold standard for diagnosis is a CT cystogram demonstrating contrast leakage from the urinary bladder, which is mostly intraperitoneal [4].

There are few details in the literature on the optimal management approach in these cases, as they are rarely reported. However, the management guidelines for intraperitoneal bladder injuries can be adapted for these cases. We have two management options: conservative versus surgical. The choice of management option depends on the time of diagnosis: intraoperative versus postoperative. For intraoperatively diagnosed injuries, the urinary bladder should be repaired either laparoscopically or via an open approach, according to available expertise. For postoperative diagnosis, it depends on how severe the urinary bladder injury is and if the patient has an abdominal drain left postoperatively for complex appendicitis. A conservative approach would be with a Foley catheter for seven to 10 days. For cases identified postoperatively with no drains in situ, the same approach should be used as for those diagnosed intraoperatively by diagnostic laparoscopy: washout and drainage of leaked urine, followed by surgical repair of the bladder, with a catheter in situ for seven 10 days. Both approaches would require cystography or CT cystogram before catheter removal to confirm that the urinary bladder injury has fully healed.

## Conclusions

Laparoscopic-induced urinary bladder injuries, if not identified early and managed promptly, may lead to significant morbidity. Reducing their incidence may be achieved by using an appropriate site for suprapubic trocar insertion, guided by laparoscopic direct vision; ensuring adequate bladder emptying; and possibly adapting routine Foley catheter use in anticipated complex cases.

Removal of ports under vision and monitoring of patient symptoms and signs in the first 24 hours postoperatively can help diagnose these cases early on. Monitoring the amount and contents of the drain output, and assessing the creatinine level of the suspected drain fluid, can be the first signs to suspect and diagnose such injuries. The gold standard for diagnosis is CT cystography; if diagnosed, the injury can be managed conservatively or surgically, depending on the factors mentioned above. This is to be followed by imaging before catheter removal.

We recommend a further literature review and the conduct of more robust studies to provide strong evidence on risk factors and the optimal approach to managing these cases.
